# A non-zircon Hf isotope record in Archean black shales from the Pilbara craton confirms changing crustal dynamics ca. 3 Ga ago

**DOI:** 10.1038/s41598-018-19397-9

**Published:** 2018-01-17

**Authors:** Yona Nebel-Jacobsen, Oliver Nebel, Martin Wille, Peter A. Cawood

**Affiliations:** 1 Isotopia Laboratory, School of Earth Atmosphere and Environment, Monash University, 9 Rainforest Walk, VIC 3800 Clayton/Melbourne, Australia; 2 Department of Geoschience, University of Tübingen, Wilhelmstraße 56, 72076 Tübingen, Germany; 3 Institute of Geological Sciences, University of Bern, Baltzerstrasse 1-3, 3012 Bern, Switzerland

## Abstract

Plate tectonics and associated subduction are unique to the Earth. Studies of Archean rocks show significant changes in composition and structural style around 3.0 to 2.5 Ga that are related to changing tectonic regime, possibly associated with the onset of subduction. Whole rock Hf isotope systematics of black shales from the Australian Pilbara craton, selected to exclude detrital zircon components, are employed to evaluate the evolution of the Archean crust. This approach avoids limitations of Hf-in-zircon analyses, which only provide input from rocks of sufficient Zr-concentration, and therefore usually represent domains that already underwent a degree of differentiation. In this study, we demonstrate the applicability of this method through analysis of shales that range in age from 3.5 to 2.8 Ga, and serve as representatives of their crustal sources through time. Their Hf isotopic compositions show a trend from strongly positive εHf_initial_ values for the oldest samples, to strongly negative values for the younger samples, indicating a shift from juvenile to differentiated material. These results confirm a significant change in the character of the source region of the black shales by 3 Ga, consistent with models invoking a change in global dynamics from crustal growth towards crustal reworking around this time.

## Introduction

The onset of plate tectonics, associated crustal evolution, and related changes in crustal chemistry are strongly debated e.g.^1–3^. Estimates for the initiation of plate tectonics range over 3 billion years from the Hadean to Neoproterozoic^4^. This uncertainty reflects the fragmentary and incomplete nature of the rock record, as well as differences in criteria and datasets used to characterize plate tectonic activity e.g.^5,6^. Most studies have been based on a comparison between rock associations from modern plate tectonic environments with those from ancient successions, notably through geochemical and isotopic data, highlighting similarities and/or differences. Recent geochemical studies of Archean crustal domains have shown that their bulk composition records a change from a predominantly mafic tholeiitic to a more evolved calc-alkaline character between 3 Ga and 2.5 Ga e.g.^7,8^. This shift in crustal chemistry has been linked to changes in global geodynamics, most likely associated with the onset of subduction^8,9^.

In this paper, we document the Hf isotopic characteristics of black shales from the Pilbara craton of Western Australia, establishing the applicability of this approach in recording the character of changes in crustal source character. These rocks display a change from a juvenile to an evolved crustal source by ca. 3 Ga. In conjunction with field and geochemical data from other sites, we suggest this reflects the development of sufficient crustal rigidity to enable widespread subaerial exposure of the source region and is consistent with models invoking the initiation of subduction of lithospheric plates at this time.

## Approach

A key problem in assessing crustal evolution is the sparse rock record of the Archean. Of today’s accessible crust, only ca. 7% is older than 2.5 Ga^6,10^, which is also often heavily deformed and chemically altered. A way to circumvent this issue is the analysis of ancient sediments derived from these early crustal assemblages. These rocks preserve a representative chemical composition of their source regions, even when the source is no longer preserved e.g.^11^.

Hafnium isotopes are an excellent tracer of crust-mantle evolution and changes in bulk crustal chemistry, because of their time-integrated parent-daughter ratio evolution (^176^Lu/^177^Hf), which reflects partial mantle melting processes as well as intra-crustal reworking e.g.,^12^. Thus, Hf isotopes from mineral archives, such as zircons, are a popular method to provide a window into Earth’s early crustal evolution. Their robustness against weathering and re-melting together with single grain dating, host-melt records of O isotope signatures, and their possibility to ‘freeze’ the Hf isotopic composition of their source rock, due to the extreme low Lu and high Hf concentrations, make them ideal reservoirs to study past crustal evolution e.g.^13–15^.

The Hf isotopic composition of detrital zircons is thus an extremely powerful tool to investigate sediment provenance, source rocks and evolution of lost crustal domains^16,17^. Zircons, however, form predominantly in evolved melts. More primitive, mafic melts that dominate juvenile crustal reservoirs, often do not reach Zr saturation^18^, yet constitute a substantial part, if not the majority of the Archean crustal rock record. Furthermore, short-lived crustal domains may not be accounted for in the zircon record^19^. Hence, although, zircon forms an invaluable crustal archive, its requirement with respect to specific host magma composition introduces a potential bias and is best complemented by a mafic counterpart. Unfortunately, no such *mineral* archive with low Lu/Hf and high U/Pb exists for mafic rocks.

To circumvent this issue, we investigated a series of Archean black shales with known ages between 3.46 and 2.74 Ga, overlapping the inferred time of changes in global geodynamics. Black shales are fine-grained, sedimentary rocks accumulating in low energy environments that are representative of the greater crustal provenances from which they were derived. Black shales are composed of authigenic and detrital components. Whilst signatures of aquatic mobile elements, stored in the former component, have been used track changing seawater chemistry under changing redox conditions, e.g.^20^, their detrital component has been less subject of scientific investigations.

## Geology

The Pilbara Craton of Western Australia hosts a series of Archean greenstone terranes that are composed of volcano-sedimentary sequences, including komatiites, tholeiitic basalt-rhyolite series, volcanoclastic sedimentary rocks, banded iron formations and black shale horizons^21^. These sequences are intruded by igneous rocks of the tonalite- trondhjemite-granodiorite (TTG) series and post-collisional granites. We analysed 19 samples recovered from drill cores from four different black shale horizons, covering a time span of ca. 800 Ma, between 3.5 and 2.7 Ga. Drilling was performed during the course of the Archean Biosphere Drilling Project (ABDP), and cores are stored at the Geological Survey of Western Australia in Perth. Detailed sample descriptions for each drill core are provided by^20^, who analysed their elemental and Mo-Cr isotope composition, as part of a study into paleo-oxygenation of the Archean atmosphere.

The oldest unit sampled is the 3.47 Ga Duffer Formation, Warrawoona Group, which consists of volcanic flows, pillow basalts, and sedimentary rocks, as well as a ~200 m thick black shale unit^22^. The 2.94 Ga Nullagine Group is part of De Grey Supergroup, which crops out across the entire Pilbara Craton. It represents a siliciclastic succession of a mostly deep-water sedimentary environment. The 2.77 Ga Mt Roe Basalt of the Fortescue Group consists of carbonated sedimentary rocks and interbedded flood basalts with interstratified black shale horizons. The 2.67 Ga Hardey Formation, which directly overlies the Mt Roe Basalt, is the youngest unit sampled and consists mostly of sedimentary rocks, including conglomerates, sandstones, and shales.

## Analytical Methods

Hafnium isotope analyses were performed at the Research School of Earth Sciences, Australian National University, Australia. Approximately 100 mg of de-carbonated sample material was spiked with a ^176^Lu-^178^Hf enriched mixed isotope tracer and dissolved in a HNO_3_/HF mixture in Teflon® vials. Samples were subjected to both “soft” and “hard” dissolution to evaluate the possible effect of detrital zircons on Hf values. Soft dissolution employs a chemical procedure that ensures that no zircon is dissolved and follows the method and rationale of^23^, whereas hard dissolution will dissolve any detrital zircon. Previous Mo-Cr isotope analyses of these rocks revealed a substantial detrital component^20^, which could include detrital zircon. Since the aim of our study is to obtain a representative record of bulk crustal evolution, it is important to exclude effects of detrital zircons as these will bias results towards their specific crustal end-member compositions. To evaluate the potential of detrital zircon and then to circumvent potential effects, we applied a soft-dissolution technique to the bulk sediments. This technique does not attack or dissolve zircon crystals and avoids the high pressure-high temperature dissolution step that is commonly applied to zircons. The same method has successfully been applied to partial garnet dissolutions^23^ and to mafic rocks from a layered intrusion^24^. Whilst high-pressure dissolution included zircon-derived Hf in the analyses, the ‘table top’-dissolution technique did not. For soft dissolution, vials with spiked samples were placed at 120 degrees on a hotplate for 48 hours in an HF-HNO_3_ mixture. Residues were centrifuged prior to further handling to ensure removal of any detrital phase, in particular zircon, that could be in the sample. For standard high pressure-high temperature dissolution, sample powders are placed at 200 °C in Teflon® vials inside metal jacked autoclaves for 48 hours. After dissolution and evaporation to dryness, all analysed samples were subsequently re-dissolved and dried down three times in concentrated nitric acid, and finally equilibrated with hydrochloric acid. Hafnium and Lu were separated from the rock matrix using LN-Spec® chromatography^25^. Isotope ratios were measured on a ThermoFisher Scientific® NeptunePlus ICP-MS, using a dry plasma with a Cetac® Aridus II desolvating system and reported relative to JMC-475 ^176^Hf/^177^Hf = 0.282160. Procedural blanks were 25 pg and 15 pg for Lu and Hf, respectively.

Comparison of hard vs. soft dissolution for the same samples indicates that zircons have contributed to the bulk Hf budget, albeit only in small proportions (see Table S1). The ε_Hf_(t) were calculated in the same way compared to those of the soft-dissolutions, and resulted in slightly lower values for the high-pressure batches and is interpreted here as addition of non-radiogenic Hf isotopic compositions of zircons to the Hf in the rock matrix. Soft-dissolution bulk-rock analyses are thus not compromised by detrital zircons.

Initial ^176^Hf/^177^Hf was calculated by assuming the same absolute age for all samples of each stratigraphic group (3467 Ma for Duffer Formation, 2940 Ma for Nullagine Group, 2775 Ma for the Mt Roe Basalt, and 2760 Ma or Hardey Formation samples) and the Lu decay constant from^26^ (1.867 E-11).

## Results

All black shale samples show very low Lu and Hf concentrations (<1 ppm, and 0.2–2.6 ppm, respectively), which is typical for sedimentary rocks containing a substantial chemical, authigenic component^27^. The ^176^Lu/^177^Hf are relatively uniform within samples <3 Ga from Nullagine, Mt Roe Basalt and Hardey Formation, ranging from 0.0096 to 0.014. These values are similar to the mean value for shales (0.016)^13^. Samples from the Duffer Formation, however, show considerable spread from 0.00623 to 0.03513, with elevated values typical for more mafic rocks. Samples from Nullagine, Mt Roe Basalt and Hardey Formation have initial Hf isotope values ranging from ε_Hf_(t) −5.2 to −0.2 (Table S1). The values of the Duffer Formation show significantly larger spread in initial Hf isotopic compositions, as well as overall lower values, compared to the other three units (from 0.280541 to 0.280764), in line with the wider spread in Lu/Hf. The initial Hf isotope compositions, when compared to CHUR, display highly positive values that are, in part, higher than the depleted mantle at time of formation.

The incompatible nature of rare earth elements (REE, including Lu) and the high field strength elements (HFSE, including Hf) during partial melting and associated concertation in crustal rocks together with low solubility in seawater indicate that both, Lu and Hf, are fully represented by the detrital component in the shales. The Hf isotopic compositions of weathered material can be significantly different to their continental source rocks^27^. This is the result of either preferential weathering of high Lu/Hf phases, e.g., in phosphates, or Aeolian sorting of low Lu/Hf zircons with an associated bias away from representative crust in their respective Hf isotope composition. Weathering or Aeolian sorting, or a combination of both, can contribute to the bulk chemical budget.

**Figure 1:**
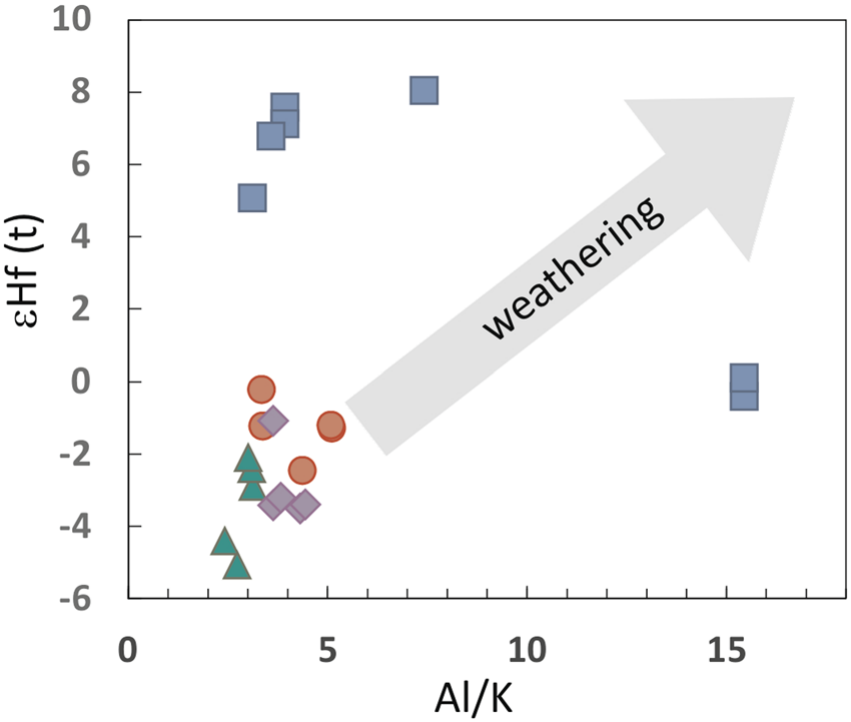
Relationship between εHf and Al/K (as an index for weathering) of black shale samples shows no correlation. Colours as in Fig. 2.

To assess the respective effects, comparison with modern sediments is appropriate. In modern sediments, progressive maturation of a sediment, that is an intensification in this process, is reflected through higher ^176^Lu/^177^Hf^28^. However, Lu/Hf in black shales from this study are low, and continental sources with a high phosphate input are improbable components prior to 2.5 Ga^29^. The so-called ‘zircon-effect’ in sediment with inherited low ε_Hf_ at the time of deposition^13^ is eliminated by the selective dissolution (‘soft dissolution’) applied in this study. It is thus concluded that only non-zircon, detrital components are contributing to the analysed batches in this study. A second effect that may compromise Lu-Hf studies in shales is that of isotope disturbance through weathering and post-depositional alteration. Studies on African sediments have shown that Al/K (as an index for weathering) and εHf are strongly correlated, trending towards more radiogenic Hf values with higher intensity of chemical weathering^30^. No such co-variation is observed in black shales of this study with both low Al/K (2.4 to 5.8) and ε_Hf_(t) (Fig. 1) leading to the conclusion that weathering had no influence on isotope systematics.

## Discussion

The epsilon notation for Hf isotope compositions allows a direct comparison of samples with different ages. With the assumption that the earliest primitive mantle was (near-)chondritic e.g.^31^, crustal Hf isotopes evolve towards negative values whereas residual mantle with higher Lu/Hf evolves towards positive values. Indeed, all values of the black shales in this study with ages <3 Ga show negative values, indicating evolved crustal domains in their provenance. The effect of Hf hosted in zircon is minimal in these samples, evidenced through the comparison of soft vs hard dissolution. It is, however, noteworthy that this effect on Hf isotopic compositions towards unradiogenic Hf, if present, would be even larger if zircon contributed more to the bulk sediment. Crustal formation model ages, using an average ^176^Lu/^177^Hf = 0.0093 for evolved rocks, of these samples all add up to a peak at ca. 3.25 Ga. This indicates a common provenance of sediments <3 Ga, i.e., they have been derived from similar crustal domains or even a single terrane, and/or from a different one that formed contemporaneously.

**Figure 2:**
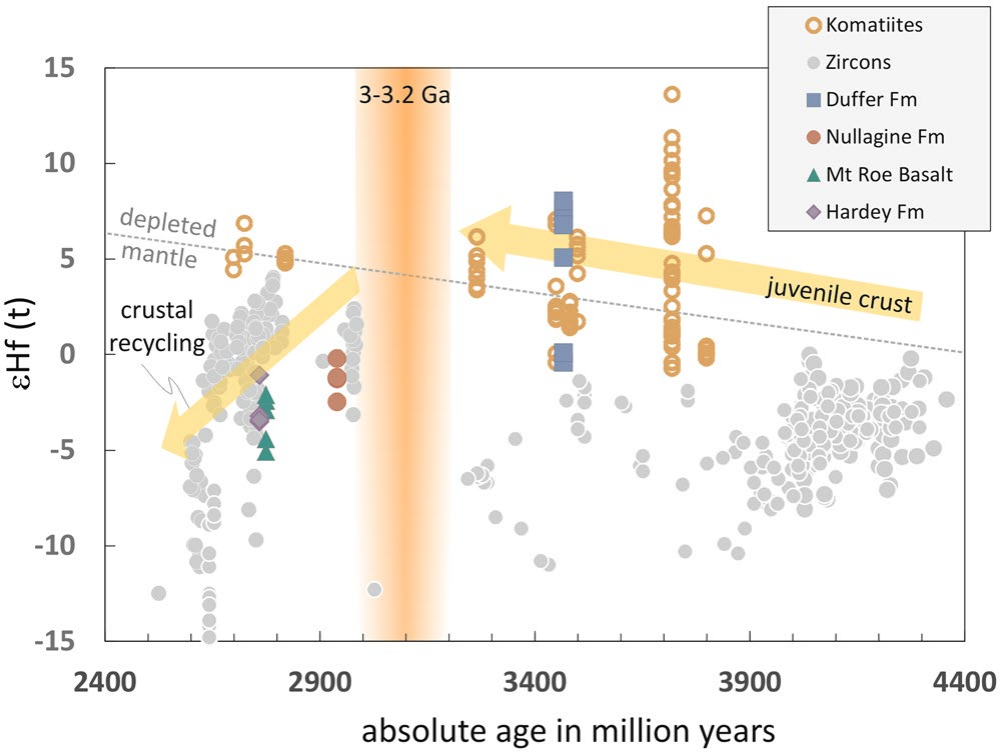
The εHf_initial_ vs absolute age plot of the black shale samples, compared to zircon^47,48^ and komatiite^49–51^ data. It shows komatiites as potential source for the older Duffer Formation. Younger Pilbara black shales show lower initial Hf. Black shales older than 3–3.2 Ga show the predominant process of formation of juvenile crust, whereas younger samples indicate crustal reworking as the dominant process. From this data set, the change in the processes can be dated to be not older than 3.2 Ga.

Samples >3 Ga do not coincide with their younger counterparts, and are systematically more radiogenic. In an ε_Hf_(t) versus time diagram (Fig. 2), they plot above the assumed ‘depleted mantle’ array, which reflects the linear evolution of the present day depleted mantle through time. The approach of describing the evolution of the whole of the depleted mantle in one single straight line is debatable, because it assumes an Earth-wide, uniform reservoir with a constant process of depletion over the past 4.4 Ga e.g.^32^, but to allow for comparison with other data, this approach is adopted here. The super-depleted, positive values indicate that the 3.5 Ga old sedimentary rocks have a provenance that is controlled by a domain of substantially more depleted, juvenile material.

Very few mid-Archean Hf isotope compositions have been reported, and data for komatiites from Barberton, Abitibi and the Pilbara, as well as rare boninitic rocks from Isua, are the only rocks of comparable age that show a similar spread and extremely positive Hf isotope composition^12,31^. Data from the literature on different komatiites show very similar εHf _initial_ to the Duffer Formation. It is therefore most plausible that komatiites contributed to substantial proportions to the detrital component in these black shales, backed by higher Cr concentrations^20^. This is important as it is the first independent evidence for the validity of extreme Hf isotopes in the early Earth other than komatiites. Figure 2 shows that komatiites plot very close, or above the depleted mantle curve, whereas zircons plot below. This is because zircons need more “Zr rich”, evolved magmas to crystallize.

It is noted that, despite being derived from one or more sources that formed at or slightly after the Duffer formation, shales studied here with depositional ages <3 Ga have been sourced from evolved, reworked crust. This crust had little, if any juvenile input. Since shales source an area potentially larger than single river systems, this may have been so, on average, for an entire Archean terrane.

## Implications and Conclusions

**Figure 3:**
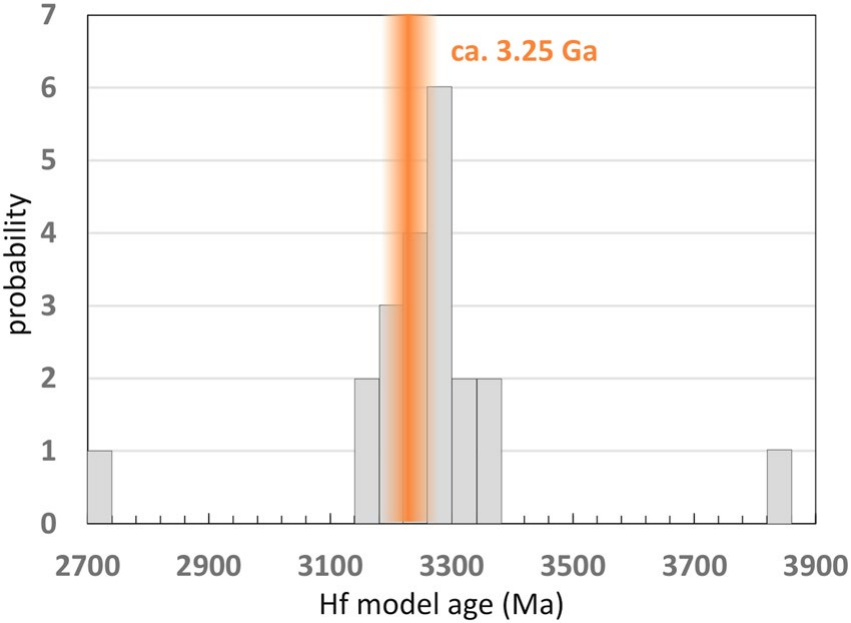
Probability plot of the Hf model ages for the Pilbara black shale samples. The peak around 3.25 Ga indicates a common source region for all samples.

The oldest black shales sampled (Duffer Formation) were derived from mafic, most likely komatiitic rocks, as indicated by varying Lu/Hf and super-chondritic initial ε_Hf_(t) (Fig. 2). In this era of the Earth’s history, formation of new crust was a dominant process and at least ~65% of the present day volume was present, as calculated by^7^, and was dominated by komatiitic assemblages. This crust seemingly lacked exposure of evolved rocks that upon weathering would have fed the sedimentary sequences of the black shales. In contrast, the <3.0 Ga samples of black shale display a change in the character of the crustal source terrane as preserved in Hf isotope signatures, reflecting the change in time-integrated Lu/Hf in crustal domains. Pilbara black shales <3 Ga indeed record host material with low ε_Hf_(t) and crustal, time-integrated Lu/Hf. Using their Hf model ages as an indicator for the first formation of their parental domain yields a time of approximately 3.25 Ga (Fig. 3). Even though this time predates proposed global changes in average crustal chemistry, indicated at around 3 Ga, it possibly marks a time of enhanced cratonic formation that initiated this change. Integrated structural and geochemical studies of the Pilbara craton have noted a change from within-plate-like magmas and dome and basin features related to vertical tectonics prior to 3.2 Ga to magmatic rocks with subduction related signatures and thrust-dominated structures at <3.1 Ga^33,34^. Other independent data sets have also noted a transition in the character of the Pilbara crust at around this time including the change in felsic magmatic activity from TTG to K-granite and peraluminious granite^35^. On a global scale, changes in average Rb/Sr of new crust^36^, and Ni/Co and Cr/Zn in terrigenous sedimentary and igneous rocks^37^, indicate a change from more mafic to felsic crustal compositions and increases in the thickness and volume of continental crust in the period between 3.2 to 2.5 Ga. These changes are accompanied by the first records of subaerial large igneous provinces^38^ and deviations in ^87^Sr/^86^Sr isotope ratios in seawater away from background mantle values after 3 Ga^39^, and evidence for a global increase in the sedimentary contribution to the magmatic record through higher δ^18^O since the late Archean^40^. These observations suggest that significant volumes of continental crust had emerged^41–45^ and were available for surficial weathering.

Our data on the Hf isotopic composition of black shales from the Pilbara craton indicate a change from the formation of juvenile crust to more evolved crustal sources and associated intra-crustal reworking from that time onwards. The timing of this change is proposed to initiated at around 3.2 Ga based on the peak in Hf model ages of the <3 Ga old shale data.

Our approach using ‘zircon-free dissolutions’ of black shales provide evidence from a crustal source without any influence of biased material, such as for instance zircons with a low Lu/Hf. We therefore are able to provide information on pristine crustal material within the limitations of the sampling area. In combination with other global data sets, we consider this change from formation of juvenile crust to crustal reworking to reflect the subaerial emergence of significant volumes of continental crust in the Pilbara region. This change in the character of the Pilbara crust is consistent with a change in global geodynamics from a stagnant lid to a plate tectonic regime e.g.,^6,31,33,46^ and took place in response to increasing lithospheric rigidity through mantle cooling.

## Electronic supplementary material

Supplementary Information 
