# Supplementary material for: A non-zircon Hf isotope record in Archean black shales from the Pilbara craton confirms changing crustal dynamics ca. 3 Ga ago
Source: Sci Rep. 2018 Jan 17;8:922. doi: 10.1038/s41598-018-19397-9 (PMC5772043; doi:10.1038/s41598-018-19397-9)
Supplement: Supplementary file 1 — Supplementary Information [file 41598_2018_19397_MOESM1_ESM.doc]

**A non-zircon Hf isotope record in Archean black shales from the Pilbara craton confirms changing crustal dynamics ca. 3 Ga ago**

Supplementary data and information

Yona Nebel-Jacobsen* 1

Oliver Nebel 1

Martin Wille 2,3

Peter A. Cawood 1

1 Isotopia Laboratory, School of Earth Atmosphere and Environment, Monash University, 9 Rainforest Walk, VIC 3800 Clayton/Melbourne, Australia

2 Department of Geoschience, University of Tübingen, Wilhelmstraße 56, 72076 Tübingen, Germany

3 Institute of Geological Sciences, University of Bern, Baltzerstrasse 1-3, 3012 Bern, Switzerland

*Corresponding author: yona.nebel-jacobsen@monash.edu

|  | **Sample name** | **Age of formation (Ma)** | **ppm Lu** | **ppm Hf** | **^176^Lu/^177^Hf** | **^176^Hf/^177^Hf** | **εHf _initial_** | ***T (DM)*** |
| --- | --- | --- | --- | --- | --- | --- | --- | --- |
| Duffer | 593 | 3467 | 0.093 | 2.041 | 0.006226 | 0.281167±5 | 7.6 | *3259.2* |
|  | *593** |  | *0.042* | *0.457* | *0.012745* | *0.281591±20* | *7.1* | *3280.6* |
|  | 594 |  | 0.045 | 0.513 | 0.01191 | 0.281477±5 | 5.1 | *3341.2* |
|  | 595 |  | 0.105 | 0.683 | 0.021012 | 0.282134±5 | 6.8 | *3146.9* |
|  | 596 |  | 0.142 | 0.687 | 0.028399 | 0.282663±8 | 8.1 | *2709.0* |
|  | 597 |  | 0.040 | 0.156 | 0.035112 | 0.282874±22 | -0.4 | *5240.4* |
|  | *597** |  | *0.066* | *0.316* | *0.028594* | *0.282453±7* | *0.1* | *3839.7* |
| Nullagine | 598 | 2940 | 0.063 | 0.679 | 0.012795 | 0.281575±4 | -1.3 | *3260.1* |
|  | 599 |  | 0.053 | 0.581 | 0.01247 | 0.281524±6 | -2.5 | *3320.2* |
|  | 600 |  | 0.051 | 0.526 | 0.013314 | 0.281607±5 | -1.2 | *3270.3* |
|  | 401 |  | 0.064 | 0.652 | 0.013566 | 0.281649±4 | -0.2 | *3217.2* |
|  | 402 |  | 0.055 | 0.528 | 0.014242 | 0.281659±5 | -1.2 | *3283.8* |
| Mt Roe Basalt | 587 | 2775 | 0.097 | 1.145 | 0.011571 | 0.281533±1 | -2.9 | *3246.5* |
|  | 588 |  | 0.122 | 1.668 | 0.010048 | 0.281465±4 | -2.4 | *3149.2* |
|  | 589 |  | 0.179 | 2.561 | 0.009578 | 0.281365±3 | -5.1 | *3281.5* |
|  | 590 |  | 0.106 | 1.323 | 0.011035 | 0.281527±19 | -2.1 | *3199.4* |
|  | 591 |  | 0.171 | 2.382 | 0.009835 | 0.281398±4 | -4.4 | *3245.6* |
| Hardey | *403** | *2760* | *0.066* | *0.636* | *0.014154* | *0.281726±2* | *-1.1* | *3183.3* |
|  | 403 |  | 0.052 | 0.508 | 0.014032 | 0.281654±4 | -3.4 | *3265.4* |
|  | 404 |  | 0.079 | 0.854 | 0.012738 | 0.281592±4 | -3.2 | *3228.2* |
|  | 405 |  | 0.108 | 1.190 | 0.012412 | 0.281567±2 | -3.5 | *3286.1* |
|  | 406 |  | 0.120 | 1.442 | 0.011439 | 0.281519±2 | -3.4 | *3260.5* |

Table 1: Lu-Hf data of black shale samples. Asterisks mark samples that underwent a different dissolution method to include zircons (see main article for details). Errors are 2σ.

<
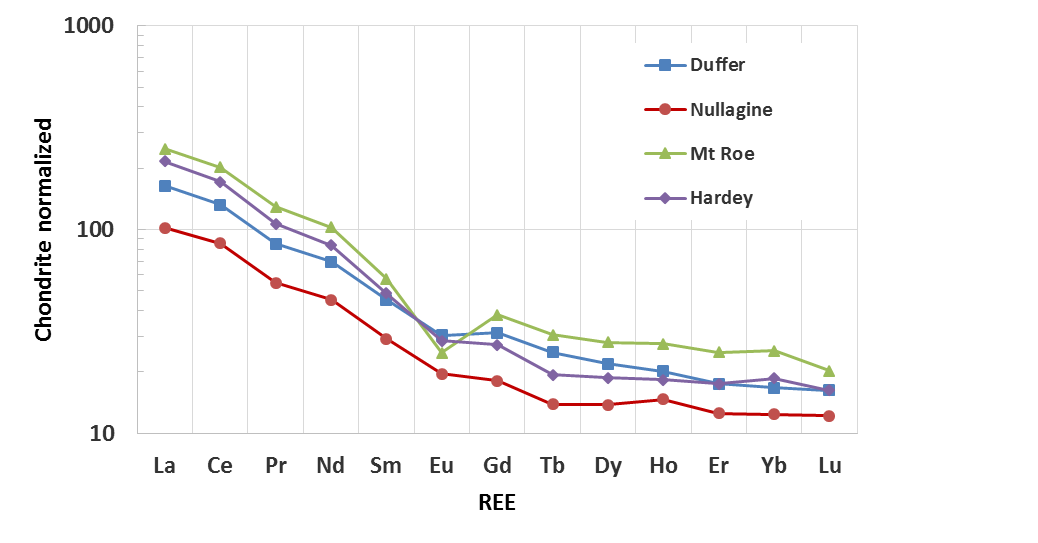


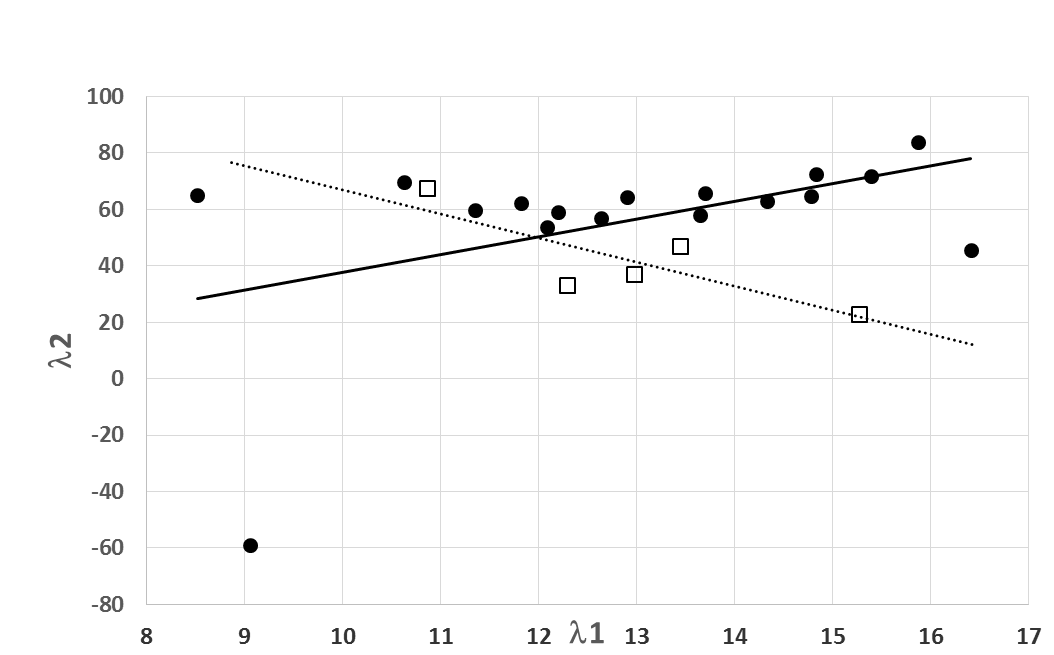
>

Figure 4a: Chondrite^1^-normalized REE pattern of the black shale units. The values are taken from^2^; these authors also modelled the different source rock contributions of the black shales, arguing that they differ significantly between the units. The suitability of REE plots for the provenance of sediments is limited ^i.e.3^. Lambda values are more suitable to demonstrate the differences in steepness and curvature of REE patterns. b: Lambda values of REE summarising the smoothness of the REE pattern^4^ (squares, dashed line: > 3.2Ga vs. dots, solid line: <2.7Ga). Lambda1 represents the steepness of REE, whereas λ2 shows the curvature. What can be observed is that the latter changed from pre- to post ca. 3 Ga.

1 McDonough, W. F. & Sun, S.-S. The composition of the Earth. *Chemical geology* **120**, 223-253 (1995).

2 Wille, M. *et al.* Mo–Cr isotope evidence for a reducing Archean atmosphere in 3.46–2.76 Ga black shales from the Pilbara, Western Australia. *Chemical Geology* **340**, 68-76 (2013).

3 He, M. *et al.* Geochemistry of fine-grained sediments in the Yangtze River and the implications for provenance and chemical weathering in East Asia. *Progress in Earth and Planetary Science* **2**, 32 (2015).

4 O’Neill, H. S. C. The smoothness and shapes of chondrite-normalized rare Earth element patterns in basalts. *Journal of Petrology* **57**, 1463-1508 (2016).
